# Unilateral Laryngeal Pacing System and Its Functional Evaluation

**DOI:** 10.1155/2017/8949165

**Published:** 2017-01-19

**Authors:** Taiping Zeng, Zhiping Zhang, Weiwei Peng, Fei Zhang, Baker Y. Shi, Fangyi Chen

**Affiliations:** ^1^State Key Laboratory of Robotics, Shenyang Institute of Automation, Chinese Academy of Sciences, Shenyang 110016, China; ^2^Department of Biomedical Engineering, Southern University of Science & Technology, Shenzhen 518055, China; ^3^University of Chinese Academy of Sciences, Beijing 100049, China; ^4^The First Affiliated Hospital of the Medical College, Shihezi University, Shihezi, China; ^5^Brain Function and Psychological Science Research Center, Shenzhen University, Shenzhen, China; ^6^Children's Hospital of Zhengzhou, Zhengzhou, China; ^7^Metokos LLC, P.O. Box 219244, Portland, OR 9221, USA

## Abstract

*Goal*. To establish a reliable instrumental system for synchronized reactivation of a unilaterally paralyzed vocal fold and evaluate its functional feasibility.* Methods*. Unilateral vocal fold paralysis model was induced by destruction of the left recurrent laryngeal nerve (RLN) in anesthetized dogs. With a micro controller-based electronic system, electromyography (EMG) signals from cricothyroid (CT) muscle on the ipsilateral side were recorded and used to trigger pacing of paralyzed vocalis muscles. The dynamic movement of vocal folds was continuously monitored using an endoscope, and the opening and closing of the glottis were quantified with customized imaging processing software.* Results*. The recorded video images showed that left side vocal fold was obviously paralyzed after destructing the RLN. Using the pacing system with feedback triggering EMG signals from the ipsilateral CT muscle, the paralyzed vocal fold was successfully reactivated, and its movement was shown to be synchronized with the healthy side.* Significance*. The developed unilateral laryngeal pacing system triggered by EMG from the ipsilateral side CT muscle could be successfully used in unilateral vocal fold paralysis with the advantage of avoiding disturbance to the healthy side muscles.

## 1. Introduction

Bilateral or unilateral vocal cord paralysis (VCP), implying vocal fold immobility, can present as dysphonia, loss of the upper register of the voice, hoarseness, breathiness, throat pain, choking episodes, or decreased vocal stamina [1–5]. Conventional treatments, such as vocal cord injection and various thyroplasty procedures [6–9], have been performed to reposition the immobile vocal fold back to the midline, but these treatments ignore the long term effects of muscle atrophy on vocal fold mass and position, with outcome greatly depending on the experience and skills of the surgeon. Later, attempts such as nerve-to-nerve anastomosis and neuromuscular pedicle transplantation have been made toward reinnervation and remobilization of the vocal fold with limited success [10–12]. Most approaches so far studied have failed to do justice to the dynamic profile inherent to normal laryngeal function and showed disappointing clinical applications. A more physiological treatment that restores dynamic laryngeal mechanisms for voice, swallowing, and breathing will improve treatment outcomes.

Recently, functional electrical stimulation (FES) of the paralyzed muscle, as a potential therapy for restoring function of a denervated muscle system, has been debated as an innovative treatment in the management of patients with laryngeal paralysis [13, 14]. Numerous studies in acute and chronic animal models have demonstrated that FES restores mobility of paralyzed laryngeal muscles [1, 12–16]. Most of these studies have focused on pacing of vocalis muscles for bilateral VCP; for example, electrical stimulus to the paralyzed posterior cricoarytenoid (PCA) muscles in human larynx has induced vocal fold abduction and restored ventilation through the glottis in case of bilateral laryngeal paralysis [17–19]. Kojima et al. and later Goldfarb et al. [20, 21] electrically evoked adduction of a unilaterally paralyzed vocal fold using electromyogenic (EMG) signals from the cricothyroid (CT) and vocalis muscles contralateral to the paralyzed vocal fold (the healthy side) as the trigger. However, recording the EMG from the healthy side muscles may introduce side effect of possible damage to the healthy side vocal fold.

Thus, the present study aims to examine the utility of EMG from surviving ipsilateral laryngeal muscles as the trigger signal for pacing the paralyzed vocal fold. Specifically, VCP will be resulting from recurrent laryngeal nerve (RLN) injury that can cause impairment of both abductive and adductive functions of the vocal folds [22], and electrical pacing will be delivered on vocalis muscles with EMG feedback signals from the ipsilateral CT muscle, which is supplied by the superior laryngeal nerve (SLN) and remains unaffected when RLN is injured, and can continue generating EMG signals that can be used for pacing purposes [1, 23]. The displacement of vocal fold will be compared among baseline (without injuries), RLN injury, and RLN injury with electrical pacing using feedback EMG signals.

## 2. Materials and Methods

### 2.1. Animals

A total of 10 young dogs weighing about 10 kg were used in this study. Animals were housed in cages under temperature- and humidity-controlled conditions with a 12 h light-dark cycle (08:00–20:00 lights on) for at least 1 week before surgery, with food and water supplying ad libitum. All surgical and experimental procedures were carried out in accordance with the Institutional Animal Care and Use Committee of South University of Science & Technology of China.

### 2.2. General Surgical Procedures

**Figure 1:**
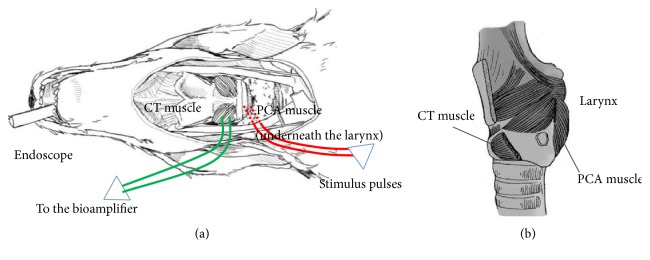
Schematic drawing of the surgical preparation. (a) Green wire shows the site where electrodes were inserted into the CT muscle to record the EMG signals. Red wire shows the site where the stimulus pulses were delivered into the PCA muscle. The dashed line section indicates that the wire is underneath the larynx. (b) The locations of CT muscle and PCA muscle.

Prior to the surgery, animals were anesthetized with 3% sodium pentobarbital (1.5 ml/kg, i.p.). Supplementary doses (a quarter of the original) of sodium pentobarbital were given when necessary to maintain anesthesia during surgery. The carotid sheath was opened to expose vagus nerves bilaterally. The RLN, as well as CT muscle, were exposed in the tracheoesophageal groove on both sides. As shown in Figure 1, electrodes were inserted into the CT muscle for recording and amplifying of the EMG signals (green wires in Figure 1), while another pair of electrodes were inserted underneath the larynx to stimulate the PCA muscle (red wire in Figure 1). A 0-degree rigid endoscope was orally advanced into the laryngeal vestibule. The scope was equipped with a CMOS camera at the tip, which would record video images and transfer them back to a PC via a USB connector. A software package on the PC controlled the start and end of the recording.

### 2.3. Pacing Instrument System

**Figure 2:**
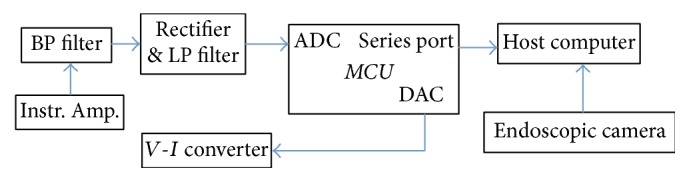
Schematic diagram of pacing instrument system.

An electronic system was built to record the EMG signals and deliver the stimulus pulses. The diagram of the system is shown in Figure 2. The core of the system is a micro controller (STM32F103RC). A bioamplifier front end was implemented with an instrument amplifier INA333 (Texas Instruments) and filter circuit. The gain of the system can be set from 100 to 5000 folds and the pass-band of the filter is 10 Hz–1500 Hz [24]. The amplified signal was rectified and low-pass filtered to extract its envelope, which was fed into the analog-to-digital converter (ADC) in the Micro Controller Unit (MCU). The MCU compares the digitized EMG envelop with a preset threshold to determine whether to trigger the stimulus. If above the threshold, the MCU generates a sequence of electrical pulses with adjustable pulse-width, period, and amplitude with its digital-to-analog converter. This pulse train was then fed into an integrated voltage-to-current converter XTR111 (Texas Instruments) to generate the current pulses to apply to the target muscle.

Needle electrodes (Medtronic Xomed Inc., Jacksonville, FL) were used to both record the EMG and deliver the stimulus. Additionally, the MCU system with a series port could communicate with a computer via a USB series adaptor. The host computer running on custom software developed in MATLAB could save the EMG data and coordinate with the video taken with the endoscopic camera.

### 2.4. Test Procedures

To test whether it is possible to induce the contraction and abduction of paralyzed vocal fold and synchronization using feedback EMG activities, we recorded vocal fold movement and CT muscle EMG during the intense breathing, when the movement of the vocal fold is obvious, according to the following steps.

1. Intact state (baseline): on the intact animal, movement of bilateral vocal folds (e.g., contraction and adduction) was recorded by the endoscope and CCD camera. EMG activities from the left CT muscle were simultaneously recorded and sent to a PC.
2. VCP model: the left RLN was crushed using forceps and then sectioned to produce left side VCP.
3. Pacing with feedback from CT muscle EMG: upon detecting feedback signals from the left CT muscle EMG activities, the pacing program activated a customized constant current stimulator to stimulate the PCA for producing vocal cord contraction. Specifically, within the MCU system, the recorded EMG signals were firstly rectified and low-pass filtered at 3 Hz to extract their envelops and then compared with a preset threshold to identify whether to trigger the stimulation. In this way, the sharp pulses in the EMG signals, due to the electrical interference or movement artifacts, can be suppressed to avoid false-trigger since its time-integration is small. Please note that the triggering threshold for individual animal was identified based on their resting-state EMG activities without spontaneous activation.

### 2.5. Data Analysis

#### 2.5.1. Measurement of Vocal Fold Displacement

**Figure 3:**
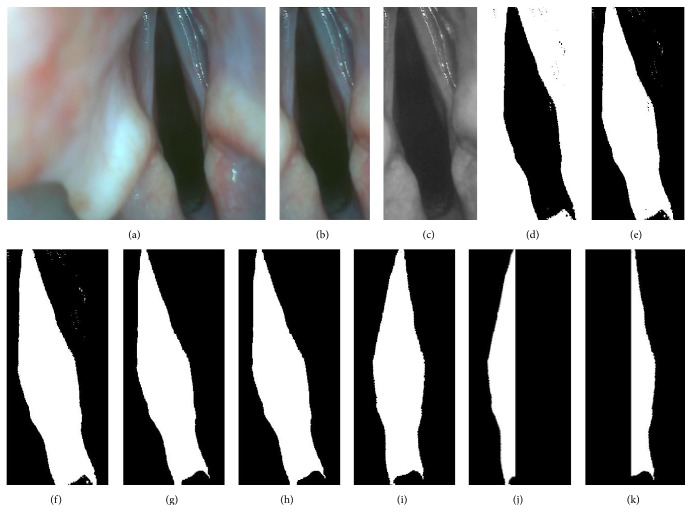
Procedure of measuring glottis movement. (a) Original image frame. (b) Extraction of glottis. (c) Grayscale image. (d) Converting of black-white image with a grayscale threshold. (e) Inverted black-white image. (f) Removing of extra black punctae within the glottis. (g) Removing of the slim link around the glottis by using image erode. (h) Removing of small island by using an area threshold. (i) Rotation of the image. ((j) and (k)) Division of left and right part of glottis.

The vocal fold displacement is quantified by measuring the area of the glottis. Image segmentation was performed on each frame of the video to automatically identify the glottis. With the application of brightness threshold and edge detection of voice cord, as displayed in Figure 3, the glottis could be extracted from the images in the MATLAB (MathWorks, Natick, MA, USA) environment according to the following steps [25].

1. The glottis, region of interest (Figure 3(b)), was firstly manually selected around the glottis from the first image frame (Figure 3(a)) of the video. This region's position was the same for every single frame on account of small relative movement between the CCD camera and the glottis in a short period of time. This process was beneficial to reduce computational complexity and avoid the disturbance of the surrounding area.
2. The colored image was converted into the grayscale image (Figure 3(c)) using monochrome images of extraction or function rgb2gray.
3. The region of interest around the glottis was binary thresholded (Figure 3(d)) and inverted (Figure 3(e)), yielding black-white glottis image. The selection of threshold used a scale factor to adapt the image intensity change due to the change of orientation and position between the CCD camera and the glottis.
4. The region of white glottis was filled with holes to remove the small black islet (Figure 3(f)), while the utility of image erode was to smooth the edges and cut the slim links around the white glottis (Figure 3(g)). Proper area threshold was subsequently applied to extract the white glottis and remove the small island (Figure 3(h)).
5. Finally, the region of white glottis was rotated (Figure 3(i)) and splitted into two parts (Figures 3(j) and 3(k)) according to the paramedian vocal fold position. The angle of rotation was determined by the two points manually inputted along the paramedian vocal fold position. With the left and right sides identified using two previous points, their areas were computed subsequently using MATLAB function of regionprop.

One of the images with biggest glottis area was firstly cropped from the recorded video, and the total number of pixels within the total glottis area was identified in Photoshop environment. As shown in Figure 3, the glottis was divided into left and right part, and the percentage of glottis area opening for each side was calculated as the ratio of the left and right glottis area to the total glottis area. In such case, the values of the left and right glottis area were normalized.

#### 2.5.2. EMG Activity Analysis

**Figure 4:**
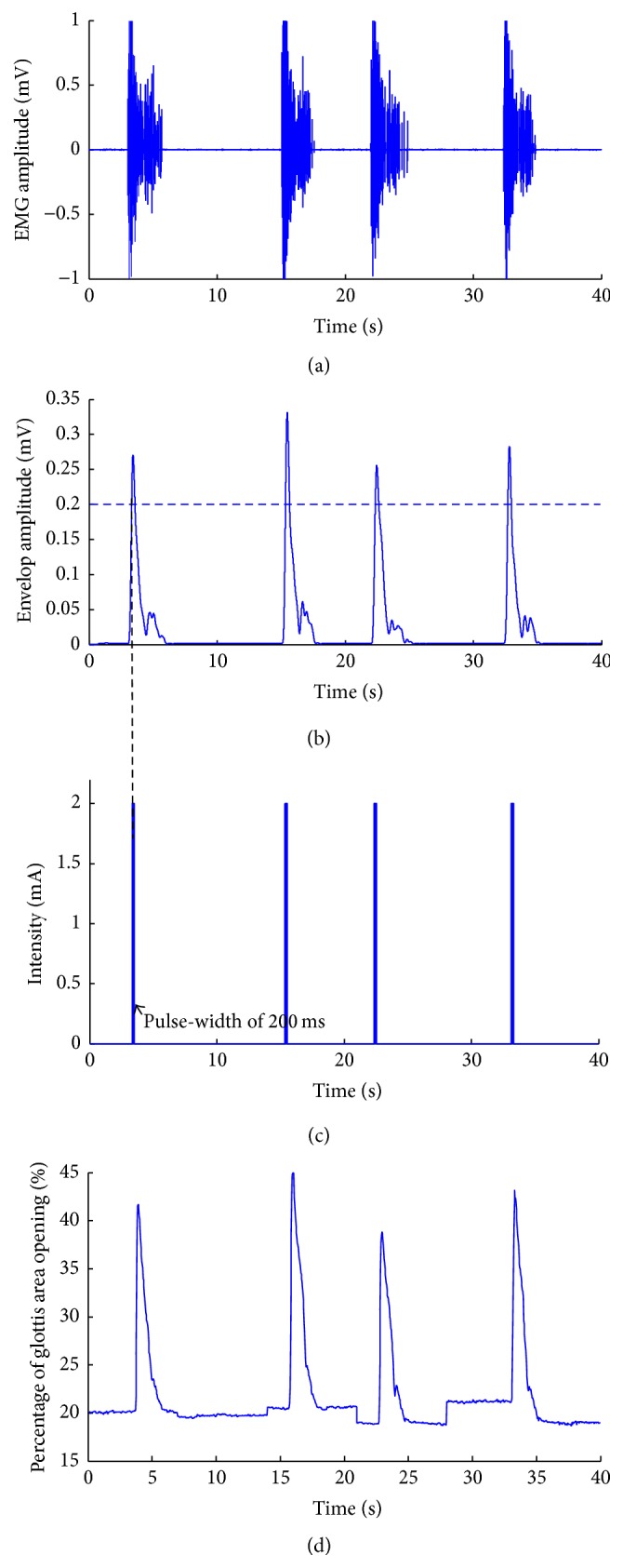
Procedure of extracting EMG signals envelope. (a) Plot of EMG amplitude changes over time. (b) Envelope of the EMG. (c) Triggered electrical stimulation, a pulse sequence with pulse-width of 200 ms, and current amplitude of 2 mA (period of 20 ms, duty-cycle varying from 50% to 100%). (d) Displacement of vocal fold across time in the intact state.

EMG activities from the left CT muscle across experimental conditions (baseline recording, left RLN injury, and pacing with feedback) were visually examined and measured. With a customized bioamplifier, the EMG signal was amplified at 1000 times and filtered with a 2nd-order band-pass filter within 10–1500 Hz. Envelops [26] of the filtered signals were extracted by full-wave rectifying and low-pass filtering at 3 Hz. The MCU sampled the EMG envelope and compared it with a preset threshold (identified individually based on the EMG activities in resting state) to determine whether to trigger the pacing system; that is, if the amplitude of calculated EMG envelopes is higher than the defined threshold, the MCU would deliver the trigger to the pacing stimulator to induce contraction/abduction by PCA muscles. Such logical judgment was based on the observation that the EMG amplitudes are higher during vocal fold movements.  Figure 4 shows the EMG triggering signal (see Video s1 in Supplemental Materials available online at https://doi.org/10.1155/2017/8949165).  Figure 4(a) shows the spontaneous EMG signal; Figure 4(b) shows the extracted envelope that will be compared with a threshold; Figure 4(c) shows the current pulses of the triggered signals.

## 3. Results

**Figure 5:**
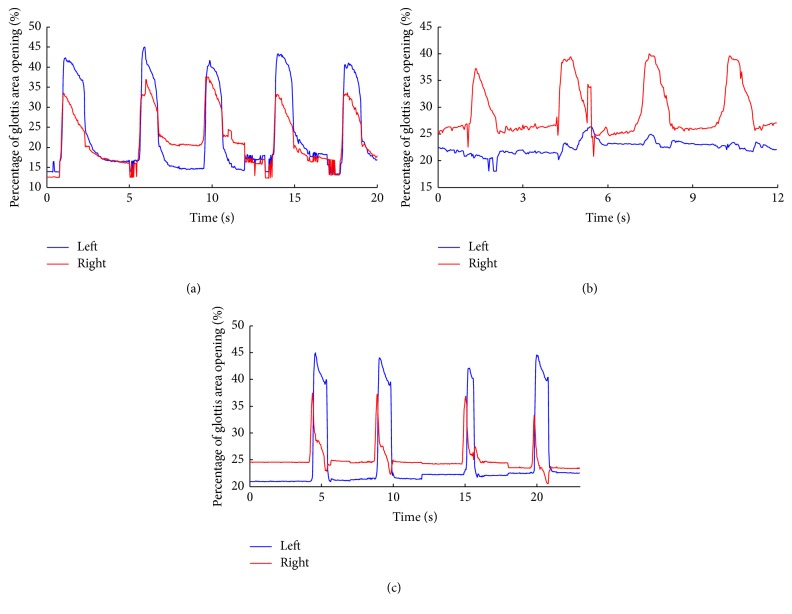
Displacement of vocal fold across experiment conditions. The movements of left (blue) and right (red) vocal fold were separately calculated. (a) Changes of glottis area in the intact state. (b) Changes of glottis area following left RLN destruction. (c) Changes of glottis area following pacing with feedback from CT EMG. The left (injured) and right (healthy) sides of glottis movements were plotted using blue and red solid lines, respectively.

Totally ten dogs were used in the experiment. Three dogs in the experiment completed all test procedures, and Figures 4 and 5 show representative results. Two dogs died from anesthesia complications and five dogs could not complete all test procedures for technical difficulties. EMG signals associated with vagal nerve stimulation-induced muscle contraction were readily recorded from the left CT muscle. Envelops of EMG signals on the single-trial level were extracted and then compared with predefined threshold to identify whether to trigger the stimulation system. In the intact state (Figure 4), the amplitudes of EMG activities from left CT muscle increased with the opening of glottis within the range of 0.3 mV–0.8 mV, indicating the synchronization between the glottis movement and change of EMG amplitudes from left CT muscle. Considering that the recorded EMG signals may be highly contaminated by the electrical pulses, once EMG signals were detected to be higher than preset threshold, one electrical pulse with fixed duration was delivered to PCA without continuous feedback from left CT muscle. In such case, the electrical pulse with fixed parameters (e.g., duration, intensity, and frequency) could be delivered without being influenced by the artifacts of EMG activities induced by electrical pacing. Thus, EMG signals from left CT muscle that could be synchronized with glottis area change in both intact and pacing states could be efficiently used as reliable feedback signals for electrical pacing.

Figure 5 shows vocal folds displacement during intense breathing, which was measured by calculating the area of glottis for each experimental condition.  Figure 5(a) shows the intact state (see Video s2 in Supplemental Materials), when the left and right vocal folds move synchronously. Due to the relative movement and orientation changes between the CCD camera and the glottis, it was difficult to identify the midline, and thus the magnitudes of areas were not precisely measured.  Figure 5(b) shows the destructed state with left RLN injury (see Video s3 in Supplemental Materials), when left (injured) side of vocal fold showed no obvious movement.  Figure 5(c) shows the displacement with electrical pacing over left PCA using feedback EMG signals from left CT muscle (see Video s4 in Supplemental Materials), when left (injured) side of vocal fold movements could be clearly seen in synchrony with the healthy side, indicating the validity of functional pacing using the EMG signals from the left CT muscle (following ipsilateral RLN destruction). It should be noted that movement onset difference between left and right side of vocal fold could be identified by careful observation, which is quite likely due to the filtering delay and the process of the envelope signal. Such onset delay (about 0.1–0.5 s) could be further tested on the single-trials with effective feedback. The synchronization of vocal fold movement between left and right sides (Figure 5(c)) indicates that such EMG envelope could be used as a reliable triggering feature for laryngeal pacing.

## 4. Discussion

The present study attempted to remobilize the paralyzed vocal cord with the use of laryngeal electrical pacing with feedback triggering EMG signals from the ipsilateral CT muscle. With the monitoring of CT EMGs as well as objective measurement of displacement of vocal fold across the test conditions, we demonstrated the validity of electrical pacing of paralyzed vocal fold with feedback from ipsilateral CT muscle in unilateral vocal cord paralysis.

Here, CT muscle that is relatively easy to access was selected as the trigger source to monitor the on-off movement of the glottis. An important advantage of using ipsilateral side CT muscle as the source of triggering signals is to avoid possible side effects of disturbing or injuring the healthy side neuromuscular structures. The CT muscle in dogs is innervated by the cranial laryngeal nerve (equivalent to the superior laryngeal nerve in human). With the presence of RLN damage, stimulation at the level of the main trunk of the vagus nerve continues to activate the CT muscle on the ipsilateral side, which generates EMG activities. This is clearly demonstrated by continuing presence of EMG signals from the ipsilateral CT muscle after RLN sectioning, although with a decrease of amplitude. Please note that the decrease of EMG amplitude may be due to loss of the contribution by adjacent vocalis and other muscles supplied by the RLN in the recorded EMGs. To improve the signal to noise ratio of the recorded EMG, the envelopes of EMG activities were extracted as trigger signals for electrical pacing (Figure 4). By examining the amplitudes of EMG envelope in intense breathing, a threshold was individually defined to discriminate respiration, with a range of 0.1 mV–0.3 mV, thus determining whether or not to trigger the pacing system. The changes of EMG activities from left CT muscle and glottis area change were synchronized in both intact state and electrical pacing conditions.

The left and right sides of vocal fold movements were further evaluated by measuring vocal fold displacement (Figure 3), which showed the synchronous movement between paralyzed and intact side of the vocal cord. The on-off movement of bilateral vocal folds was synchronized in the intact condition (Figure 5(a)), and such synchronization was disrupted by left RLN injury (Figure 5(b)), that is, no movement of left side vocal fold, indicating effective unilateral vocal cord paralysis. Importantly, when using the feedback signals of EMG envelopes from left (injured side) CT muscle, the movement of left (injured) vocal fold was restored and synchronized with the healthy side (Figure 5(c)). Please note that the EMG envelops were compared with a preset threshold (predefined individually based on spontaneous EMG activities in intact state) to determine whether to trigger the pacing system, which took individual differences of EMG amplitudes into consideration. These findings clearly show that the envelope of EMG activities from the ipsilateral CT muscle after losing RLN innervation can yield sufficient signal/noise ratios that can be easily recognized and used to trigger a pacing system for the purpose of restoring movement of the paralyzed vocal fold synchronized with the healthy vocal fold.

The findings in the current study are quite consistent with previous experiments aiming at remobilizing the PCA muscle in bilateral VCP, which generally reported promising performance [1, 27, 28]. Considering the synchronization between glottal opening movement and inspiration, trigger signals that were somehow related with inspiratory movement could be picked up as feedback to the electrical pacing system [1, 16, 29, 30], for example, EMG of the diaphragm, thoracic wall movement, elongation of the trachea, and temperature difference within respiratory cycle. While unilateral VCP is much more common than bilateral VCP, unproportionally less research efforts have been directed toward restoring dynamic functions of a unilaterally paralyzed vocal fold. We confirmed the validation of remobilizing a unilaterally paralyzed vocal cord by the delivery of electrical stimulation to the paralyzed cord triggered by action potentials of the ipsilateral surviving intrinsic laryngeal muscles.

Indeed, several limitations existed in the current study. First, a slight onset lag between the stimulated vocal cord and the intact cord, of about 100–300 ms for glottal opening, was observed. Such time lag is mainly due to the filtering delay and the process of the envelope signal and can be shortened using a more efficient MCU system. Second, besides extracting EMG envelops, efficient technical methods to reduce interference by stimulus artifacts and improve signal to noise ratios should be developed. Third, we may want to investigate the long term pacing under physiological conditions, as well as long term effects of pacing on local tissues and other side effects, for the technique to be established as a useful clinical means to treat unilateral vocal cord paralysis. And needle electrodes we used in the experiment have side effects to muscles. We would use flexible electrodes [31–33] to ensure good biocompatibility and minimal side effects in the future long term pacing. Fourth, PCA was stimulated to generate abduction (not adduction) of paralyzed vocal fold in our current experiment, which aims to achieve synchronous movement of the vocal cords. In our future experiment, lateral cricoarytenoid muscle or arytenoid muscle would be stimulated to generate adduction of paralyzed vocal fold.

In summary, an instrument system was developed to record the EMG signals from surviving ipsilateral CT muscle and deliver electrical pulses for vocal cord pacing in unilateral VCP. Endoscopic imaging system and image processing software were developed to objectively evaluate the efficiency of the pacing system. EMG activities from the CT muscle ipsilateral to a paralyzed vocal fold following RLN damage are present, recordable, and sufficient to trigger a pacing system for the purpose of inducing synchronized movement of the paralyzed cord with the healthy vocal fold. This study strongly supports the feasibility of electrical stimulation as a treatment for unilateral recurrent laryngeal nerve paralysis, which offers tremendous promise in a patient population for whom traditional surgical therapies are not optimal. Future research needs to solve the many technical/engineering challenges such as interference by stimulus artifacts as well as onset delay of the paralyzed vocal fold. A miniaturized mobile pacing system will need to be developed, which would allow studying long term pacing under physiological conditions, as well as studying long term effects of pacing on local tissues and other side effects, which is a necessary step for translation to human studies.

## Supplementary Material

The glottis movements with all test procedures are recorded by the endoscopic camera. Video s1 shows the intact glottis movement with electrodes in Figure 4.
Video s2 shows the intact glottis movement without electrodes in Figure 5(a).
Video s3 shows the injured glottis movement of vocal cord paralysis (VCP) model in Figure 5(b).
Video s4 shows the injured glottis movement with pacing in Figure 5(c).
