# Supplementary material for: Unilateral Laryngeal Pacing System and Its Functional Evaluation
Source: Neural Plast. 2017 Jan 19;2017:8949165. doi: 10.1155/2017/8949165 (PMC5288527; doi:10.1155/2017/8949165)
Supplement: Supplementary file 5 [file 8949165.f5.docx]

**Supplemental materials**

**Video s1:**

Under natural breathing, the glottis moved slowly to achieve vocal fold adduction and abduction. When the glottis movement was recorded by camera, the electromyography (EMG) signal (in Figure 4) was also simultaneously recorded from cricothyroid (CT) muscle.

**Video s2:**

Under natural breathing, the left and right side of glottis moved at the same time. Figure 5A shows changes of glottis area in both sides.

**Video s3:**

When the left recurrent laryngeal nerve (RLN) was cut, the left side of vocal fold showed no obvious movement. Figure 5B shows changes of glottis area under natural breathing and that of the left (injured) side is negligible.

**Video s4:**

With electrical pacing over left posterior cricoarytenoid (PCA) and feedback EMG signals from left CT muscles, the left (injured) side of vocal fold was successfully reactivated and were synchronized with right (healthy) side. The area changes during the experiment was extracted from s4 and plotted in Figure 5C.
